# Miquelianin and spiraeoside from *Filipendula ulmaria* mitigate α-synuclein accumulation in *C.elegans* and reduce the expression of neuroinflammatory cytokines in human microglia

**DOI:** 10.3389/fphar.2025.1720314

**Published:** 2026-02-11

**Authors:** Martina Redl, Sabrina Weisenburger, Andreas Wasilewicz, Ulrike Grienke, Martin D. Lehner, Dirk Bredenbröker, Judith M. Rollinger

**Affiliations:** 1 Division of Pharmacognosy, Department of Pharmaceutical Sciences, University of Vienna, Vienna, Austria; 2 Vienna Doctoral School of Pharmaceutical, Nutritional, and Sport Sciences, University of Vienna, Vienna, Austria; 3 Preclinical R&D, Dr. Willmar Schwabe GmbH and Co. KG, Karlsruhe, Germany; 4 Research & Development, Dr. Willmar Schwabe GmbH and Co. KG, Karlsruhe, Germany

**Keywords:** anti-aging, *caenorhabditis elegans*, geroprotective, heat shock resistance, inflammation, meadowsweet, microglia cells, Parkinson disease

## Abstract

**Introduction:**

Chronic neuroinflammation and impaired proteostasis are increasingly recognized as interconnected drivers of Parkinson’s disease (PD). Due to their pleiotropic character, multicomponent herbal remedies combining anti-inflammatory and proteostatic activity may counteract these disease-promoting processes. This study investigated a hydroethanolic extract of *Filipendula ulmaria* (FE) for its ability to modulate neuroinflammation, proteostasis, and aging-associated mechanisms relevant to PD.

**Methods:**

The effects of FE on lifespan and health span were assessed in wild-type and on α-syn::YFP fluorescence, survival and thermoresistance in transgenic NL5901 *Caenorhabditis elegans*. FE was fractionated by flash chromatography, and bioactivity was assigned to individual constituents using LC-MS dereplication and a ^1^H NMR-based biochemometric approach. Identified constituents were further examined for their capacity to reduce α-syn::YFP fluorescence and enhance thermotolerance in NL5901. To assess their relevance in neuroinflammation, all samples were examined in LPS-stimulated human microglial HMC3 cells for their effects on pro-inflammatory gene expression.

**Results:**

FE extended lifespan, improved health span and reduced α-syn::YFP fluorescence in NL5901 by up to 25% at 200 μg/mL. In HMC3 cells, FE significantly downregulated *IL-1β*, *IL-6*, and *MCP-1* expression at 100 μg/mL without cytotoxicity. Biochemometric and LC-MS analyses identified the flavonoid glycosides spiraeoside and miquelianin as key constituents, reducing the α-syn::YFP fluorescence by 32% and 35% at 200 μg/mL, respectively, while simultaneously increasing thermotolerance in NL5901 and suppressing pro-inflammatory cytokine expression in microglia.

**Conclusions:**

FE represents a promising source of neuroprotective agents targeting both neuroinflammation and proteostasis, supporting spiraeoside and miquelianin as hit candidates for preventive strategies against early neurodegenerative mechanisms.

## 1 Introduction

Parkinson’s disease (PD) is the second most common age-related neurodegenerative disorder (Zheng and Chen, 2025; Long et al., 2025), characterized by motor impairment and progressive loss of dopaminergic neurons, partly driven by pathological accumulation of misfolded α-synuclein (α-syn) (Simon et al., 2020). Growing evidence suggests that disturbances in immune and stress responses shape the course of the disease from its earliest stages (Simon et al., 2020; Zhang W. et al., 2023). Chronic activation of microglia sustains a pro-inflammatory environment that contributes to neuronal damage through the release of reactive oxygen species and pro-inflammatory cytokines (Smith et al., 2012), and the propagation of misfolded aggregates (Guo et al., 2020; Zheng and Zhang, 2021). Misfolded α-syn in turn acts as a trigger for microglial activation, creating a vicious cycle in which inflammation and protein aggregation reinforce each other (Zhang W. et al., 2023).

Current pharmacological options for PD provide symptomatic relief but fail to address underlying disease-driving mechanisms(Kim and Lee, 2025). Anti-inflammatory strategies are therefore of growing interest, as they may slow progression by disrupting the vicious loop between neuroinflammation and protein aggregation. Due to their inherent pleiotropic nature herbal remedies may have the ability to combine anti-inflammatory, antioxidant, and proteostasis-modulating activities (Upadhyay, 2021), and thus represent a pharmacologically attractive option for long-term intervention (Eckert, 2010). While certain medicinal plants have been traditionally used to support the nervous system (Eckert, 2010), most of these indications predate modern neurobiological understanding. In fact, they arose long before concepts such as protein aggregation, neuroinflammation, or other disease-driving mechanisms in PD or Alzheimer’s disease were defined. Accordingly, the potential of anti-inflammatory herbal remedies to modulate neurodegenerative processes such as proteotoxicity, protein aggregation and neuroinflammation remains insufficiently explored. Nevertheless, given their structural diversity, natural products represent a valuable source of bioactive compounds whose indications extend far beyond the ethnopharmacological context (Bernardini et al., 2018).

Among these, *Filipendula ulmaria* (L.) Maxim or meadowsweet has long been valued for its anti-inflammatory effects, consistently demonstrated across *in vitro* and *in vivo* studies (Katanić et al., 2016; Katanić et al., 2018), yet has received little attention in the context of neurodegeneration. From a phytochemical perspective, the anti-inflammatory activity can be attributed to the ubiquitous class of polyphenols, present at high levels in *F. ulmaria* extracts, including salicylates (Marks and Smith, 1960; Yin et al., 1998; Baltazar et al., 2011), other phenolic acids (Kumar and Goel, 2019), flavonoid glycosides (Dias et al., 2021), and tannins (European Medicines Agency, 2011). Previous *in vitro* studies link the anti-inflammatory potential of *Filipendula sp*. and its constituents to a distinct NO radical scavenging activity and the modulation of the arachidonic acid cascade by the inhibition of cyclooxygenase −1 and 2 (COX-1 and COX-2) (Katanić et al., 2018), two isoenzymes involved in CNS inflammation (Choi et al., 2009). Notably, *in vivo,* the anti-inflammatory potential of *F. ulmaria* could be demonstrated by a reduction of inflammation and associated pain (Samardžić et al., 2016) and the ability to attenuate oxidative damage and apoptosis in the hippocampus, restoring BDNF and GABA-A receptor levels in rat models (Arsenijevic et al., 2021). However, whether those anti-inflammatory and antioxidative properties translate into a protective effect in neurodegenerative disease contexts remains unknown.

To address this question and the associated complexity arising from the underlying pathology, we selected the nematode *C*. *elegans*, a well-established model in aging and neurodegeneration research. Indeed, as a multicellular organism with differentiated tissues and organ systems, *C. elegans* provides a biologically meaningful platform for assessing the activity of chemically complex and isolated natural products *in vivo* (Kirchweger et al., 2023). Unlike cell-based assays, it allows first insights into whole-organism parameters affecting the activity, including yet simplified intestinal absorption and oral toxicity - parameters that are highly relevant and difficult to assess *in vitro*, and captures the interplay between multiple signaling pathways. Compared to *in vivo* mammalian models, the roundworm offers a resource-efficient and ethically favorable alternative, particularly valuable in the early preclinical stage of research (Redl et al., 2024). Having the shortest average lifespan among all multicellular organisms of about 3 weeks (Zwirchmayr et al., 2020), *C. elegans* enables rapid experimental cycles while retaining a remarkable degree of genetic and physiological conservation with higher organisms (Kim et al., 2018). Simple readouts such as motility and survival under oxidative or heat stress reflect muscular integrity, systemic homeostasis, and proteostatic robustness (Rollins et al., 2017). The nematodes transparency enables direct visualization of anatomical changes and the dynamic of fluorescently tagged proteins in living animals. Owing to its genetic tractability, *C. elegans* can be engineered to express human disease-associated proteins, enabling the modeling of key aspects of neurodegenerative disorders in a simple *in vivo* model (Kirchweger et al., 2023). In particular, the transgenic strain NL5901 expresses, age-dependently, human α-syn fused to yellow fluorescent protein under control of the *unc*-54 promoter in body wall muscle cells (van Ham et al., 2008) and has become a well-established model for investigating protein accumulation in living animals (Bodhicharla et al., 2012). In NL5901, the reduction of α-syn::YFP fluorescence levels may capture reduced protein expression or accumulation (Bodhicharla et al., 2012), due to increased protein clearance, biophysical interference of aggregation or even a combination of these mechanisms.

While *C. elegans* has proven invaluable for dissecting genetic and molecular mechanisms of aging and proteotoxic stress, its utility for studying neuroinflammatory processes is fundamentally limited. The nematode lacks specialized immune cells (Pukkila-Worley and Ausubel, 2012), such as microglia, which are central to initiating and propagating inflammation in the mammalian nervous system (Gao et al., 2023). Instead, immune defense in *C. elegans* is largely confined to innate epithelial responses in the intestine and epidermis, with no direct equivalent of cytokine-driven signaling or cell mediated neuroinflammation (Pukkila-Worley and Ausubel, 2012). Consequently, inflammatory mechanisms in this model can only be assumed by indirect readouts through stress responses or organismal decline, rather than capturing the cellular and molecular complexity of neuroinflammation. These constraints require the integration of a complementary system for detailed insight into neuroinflammation.

To more specifically address aspects of neuroinflammation, the human microglial HMC3 cell model was employed in this study. The HMC3 cell line provides a reproducible and well-characterized model for studying microglial activation and cytokine secretion under inflammatory conditions. LPS stimulation of HMC3 cells leads to the upregulation of key inflammatory mediators such as Interleukin 1β (IL-1β), Interleukin 6 (IL-6), monocyte chemoattractant protein 1 (MCP-1 or CCL2), and TNF-α, mimicking the microenvironment of neurodegenerative pathology (Baek et al., 2021).

## 2 Materials and methods

### 2.1 Plant material collection

Aerial parts of *Filipendula ulmaria* (L.) Maxim. were obtained by Dr. Willmar Schwabe GmbH & Co. KG from Boletus d.o.o. (Hadžići, Bosnia and Herzegovina), batch number W300105. A voucher specimen is deposited at Dr. Willmar Schwabe GmbH & Co. KG (material number 116465550, batch W300105N1).

### 2.2 Extract generation and compounds

The *F. ulmaria* extract (FE) was prepared in the pilot plant facility at Dr. Willmar Schwabe GmbH & Co KG. In brief, dried aerial parts of *F. ulmaria* milled to a particle size of ≥ 85% ≤ 4 mm were added to 60% aqueous ethanol (w/w) and submitted to a two-phase extraction process with 2 h extraction phases at 55 °C with a drug:solvent ratio of 1:7.5. Then the primary extract solution was separated from the herbal drug material with a decanter followed by concentration to a spissum extract with a centrifugal evaporator (60 °C). Finally, the spissum extract was dried in a vacuum drying oven (60 °C, 250–20 mbar, 6 h) and milled to fine powder to yield the dry extract (DER of 3.62:1) used in the present study.

Monotropitoside (Mt), rutin trihydrate (R) and tellimagrandin I (TI) were provided by Dr. Willmar Schwabe GmbH & Co. KG. Briefly, Mt was isolated from FE through sequential liquid-liquid partitioning (using triple extraction steps each with tert-butyl methyl ether, ethyl acetate and butan-1-ol, followed by Sephadex LH-20 and RP-18 chromatography and crystallization from methanol (25%). Its identity was determined by NMR. TI was isolated from FE by liquid-liquid partitioning with tert-butyl methyl ether, ethyl acetate and butan-1-ol, followed by Sephadex LH-20 chromatography of the ethyl acetate fractions. R was previously obtained by isolation from a *Hypericum perforatum* water extract by water-butanol (17:3) extraction, followed by Sephadex LH-20 chromatography.

Other main constituents dereplicated by LC-MS from FE were obtained from commercial sources (miquelianin (Mq): BLD Pharm, CAS 22688-79-5; spiraeoside (Sp): Carl Roth, CAS 20229-56-5, quercetin (Q): Sigma Aldrich, CAS 117–39-5); the purities of all compounds used for this study were determined by ^1^H NMR to be >95%. For cell culture experiments dexamethasone was purchased from Sigma-Aldrich (Sigma #D2915). For *C. elegans* assays, 5-fluorodeoxyuridine (FUdR; BCCH8275) was purchased from Sigma-Aldrich.

### 2.3 Fractionation of FE and generation of microfractions (MFs)

1.0 g FE was fractionated using an Interchim puriFlash® system in normal phase mode, equipped with a Silica HP 220 g, 25 μm, 220 bar puriFlash column (PF-25SIHC-F0220). A stepwise solvent gradient of hexane: ethyl acetate: methanol: water was applied as follows (flow rate 26 mL/min): 0–5 min, 100:0:0:0 to 50:50:0:0; 5–10 min, 50:50:0:0 to 25:75:0:0; 10–15 min, 25:75:0:0 to 0:100:0:0; 15–35 min, 0:100:0:0; 35–55 min, 0:100:0:0 to 0:87.5:12.5:0; 55–70 min, 0:87.5:12.5:0 to 0:75:25:0; 70–80 min, 0:75:25:0; 80–95 min, 0:75:25:0 to 0:50:50:0; 95–105 min, 0:50:50:0 to 0:25:75:0; 105–120 min, 0:25:75:0 to 0:12.5:87.5:0; 120–130 min, 0:12.5:87.5:0 to 0:0:100:0; 130–140 min, 0:0:100:0; 140–145 min, 0:0:100:0 to 0:0:75:25; 145–150 min, 0:0:75:25 to 0:0:50:50. Fractions (each 15 mL) were collected over 150 min. The fractionation was repeated twice. Obtained fractions were monitored by thin-layer chromatography (TLC) using the mobile phase ethyl acetate: formic acid: acetic acid: water (100:11:11:27) after derivatization with natural product reagent and polyethylene glycol at UV360 nm. Fractions with similar TLC patterns were pooled into 19 MFs with overlapping chemical profiles.

### 2.4 UHPLC-PDA-MS and UHPLC-PDA-ELSD analysis

FE and its MF1-19 were analyzed using a Waters ACQUITY UPLC H-Class system equipped with a photodiode array (PDA) detector, an evaporative light scattering detector (ELSD), and a single quadrupole mass spectrometer (QDa) with an electrospray ionization (ESI) source. System control and data acquisition were performed using EMPOWER 3 software. Chromatographic separation was achieved on a BEH C-18 column (2.1 × 100 mm, Waters) maintained at 40 °C. The mobile phase consisted of solvent A (water with 0.1% formic acid) and solvent B (acetonitrile with 0.1% formic acid), delivered at 0.3 mL/min using the following gradient: 0 min 5% B; 3.0 min 20% B; 20.0 min 30% B; 21.0 min 30% B; 22.0 min 40% B; 24.1 min 98% B; 25.0 min 98% B; 26.0 min 5% B; 27.0 min 5% B. UV-Vis detection was performed with the PDA module across 190–600 nm. UV-Vis detection was performed using the PDA module, recording spectra across the range of 190–600 nm. Mass spectrometric detection was conducted in both positive and negative ionization modes, scanning a mass range of m/z 100–750. The QDa detector operated with a cone voltage of 15 V and a capillary voltage of 0.8 kV. For enhanced ionization, a make-up solvent composed of 10 mM ammonium formate in methanol:water (9:1, v/v) was introduced at a flow rate of 0.4 mL/min.

### 2.5 NMR analyses

Nuclear magnetic resonance (NMR) experiments were performed on a Bruker Ascend 500 MHz NMR spectrometer (Bruker, Billerica, MA, USA) operating at 298 K. The instrument was equipped with a 5 mm TCI Prodigy CryoProbe, an Avance NEO console, and a SampleJet autosampler. ^1^H NMR spectra were recorded at a resonance frequency of 500.19 MHz. Deuterated methanol was purchased from Deutero GmbH (Kastellaun, Germany). For NMR measurements, the receiver gain was fixed for each sample or group of samples with the same concentration reference to account for potential non-linearity of the receiver. ^1^H NMR spectra were acquired using a standard Bruker pulse sequence program with default settings (zg30) and 128 scans.

#### 2.5.1 ^1^H NMR spectra processing and statistical correlation with bioactivity data

^1^H NMR spectra of FE-derived MF7-9 and the reference Sp were processed with TopSpin 4.1.1, manually performing phase correction and baseline correction, applying a simple polynomial curve fitting of the mathematical equation A+ Bx + Cx^2^ + Dx^3^ + Ex^4^.

The HetCA analysis was performed in MATrix LABoratory (MATLAB) by following the procedure described in Grienke et al. (2019), with altered parameters. Briefly, the ^1^H NMR spectra were bucketed (δ_H_ 3.0–8.0 ppm) with a bucket width of 0.0005 ppm. For each bucket, the integrated ^1^H NMR resonance intensity was determined and employed as a variable in the subsequent analyses. Covariance was calculated to assess the joint variability between ^1^H NMR resonance intensities and the percentage of fluorescence reduction, expressed as % of vehicle control. The normalized form of covariance, the correlation coefficient, was also determined and used for color coding. The resulting covariance values were visualized as a ^1^H NMR pseudospectrum, with the respective color coding applied.

### 2.6 *Caenorhabditis elegans* experiments

#### 2.6.1 *C. elegans* strains, maintenance and synchronization

*Caenorhabditis elegans* wild-type var. Bristol N2, the mutant NL5901 and CL4176 and *E. coli* OP50 were obtained from the *Caenorhabditis* Genetics Center (University of Minnesota). OP50 stocks (100 mg/mL) were prepared as described in Zwirchmayr et al. (2020). All *C. elegans* strains were maintained at 16 °C on solid nematode growth medium (NGM) agar plates, seeded with an OP50 *E. coli* lawn. Worms were transferred to a new plate every week, to prevent starvation. The preparation of a synchronous worm culture was performed as outlined in Redl et al. (Redl et al., 2024), using the bleaching technique (Solis and Petrascheck, 2011). For *C. elegans* wild-type N2 and NL5901, eggs were kept in the S-complete medium without food for 48 h on a nutator at room temperature, whereas CL4176 eggs were kept at 16 °C to allow the larvae to hatch from the eggs.

#### 2.6.2 Survival and lifespan assay

Survival analysis was performed with N2 and NL5901 *C. elegans*. N2 nematodes were subjected to both survival and lifespan assays, whereas NL5901 worms were assessed only in survival assays. The protocols followed Redl et al. (2024), with one modification: NL5901 worms were treated with the samples starting at the L3 stage and kept at 20 °C during the experiment. Briefly, 3–18 age-synchronized L1 larvae were reseeded into each well of three 96-well plates and fed with OP50 *E. coli* (6 mg/mL). At the L3 stage, worms were sterilized with FUdR (0.12 mM final; Sigma-Aldrich). NL5901 larvae were additionally treated with test samples, positive control (Ctr(+)) and vehicle control at this stage, whereas N2 worms were treated at the young adult stage, on the following day. Test samples and the given positive control (Ctr(+)) were dissolved in DMSO (final concentration 0.7%) and tested in triplicate wells. 0.7% DMSO served as vehicle control. For each condition, an average of 95 worms was used (*n* ≈ 95; distributed to nine wells in total). To prevent starvation, worms were fed with OP50 on the third day of adulthood. Plates were oxygenated three times per week throughout the experiments. For survival experiments, worm viability was evaluated on day 11 of adulthood by counting the number of live worms per well. Worms were considered dead when they showed no movement or pharyngeal pumping and failed to respond to light stimuli. For lifespan experiments, survival was monitored three times per week until 50% of the population had died, defined as DT_50_. The mean survival rates and DT_50_ values from parallel replicates were plotted as bar charts using GraphPad Prism 9. Data are presented as mean ± SD. Statistical differences between treatment groups and the control group were evaluated using One-way ANOVA followed by Dunnett’s *post hoc* test. Differences were considered statistically significant at *p* ≤ 0.05.

#### 2.6.3 Body size assay

The culture maintenance and preparation followed the same procedure as described for the survival assay, according to the protocol outlined in Redl et al. (2024). Briefly, N2 *C. elegans* were synchronized and 3–18 L1 larvae were reseeded into three wells of a 96-well plate. At the L3 stage, larvae were sterilized with FUdR (0.12 mM final; Sigma-Aldrich). N2 worms were treated with the samples and the vehicle control (0.7% DMSO) at the young adult stage on the following day. After 5 days of incubation with 200 μg/mL FE, 50 μg/mL FE, or vehicle control (0.7% DMSO), two-thirds (two wells) of the worms were paralyzed with 0.2% sodium azide and both, non-paralyzed and paralyzed worms were transferred to one solid agar petri dish, each. The body length of paralyzed N2 worms was measured under a dissecting microscope using an eyepiece micrometer, calibrated with a stage micrometer (*n* = 21) (McCulloch and Gems, 2003). The body length values were presented as mean ± SD in bar charts using GraphPad Prism 9. Images of the non-paralyzed worms were acquired using a Zeiss Z1 Axio Observer inverted fluorescence microscope equipped with an Axio Cam MRm camera and a transmitted light (TL) filter. Worms were imaged under consistent settings to allow visual comparison between treatments. Statistical analysis was based on One-way ANOVA and Dunnett’s *post hoc* test.

#### 2.6.4 Motility assay

The motility assay was performed on N2 nematodes according to the protocol described by Redl et al. (2024). Nematode culture preparation and treatments were carried out as described for the survival assay using the same vehicle control and positive control. All experiments were performed in parallel triplicates. For each condition, an average of 95 worms was used (n ≈ 95; distributed to nine wells in total). Locomotor activity was measured on day 0 (basal activity) and day 7 using a semiautomatic motility tracker (WMicrotracker One, Phylumtech) for 30 min per plate. For each replicate, motility data were expressed as the mean activity of each condition relative to basal activity (%), and results were presented as bar charts (mean ± SD) using GraphPad Prism 9. Statistical analysis was assessed using One-way ANOVA followed by Dunnett’s *post hoc* test, with significance set at *p* ≤ 0.05.

#### 2.6.5 α-Synuclein assay

30–40 Age-synchronized NL5901 L1 larvae were seeded into each well of three black, clear-bottom 96-well plates and fed with OP50 *E. coli* (6 mg/mL). During the experiment, plates were kept at 20 °C. At the L3 stage, worms were sterilized with 0.12 mM FUdR (Sigma-Aldrich) and treated with test samples at respective concentrations, vehicle control (0.7% DMSO), and positive control (2.5 mM levodopa; (Ctr+))- with three technical replicate wells per condition and plate. This resulted an average of 315 worms used for each condition (n ≈ 315, distributed to nine wells in total). Basal YFP fluorescence levels of each well were measured immediately after treatment under identical acquisition settings, using a Tecan Spark microplate reader (excitation 450 nm; emission 535 nm). After 5 days of incubation, YFP fluorescence was reassessed under the same conditions and normalized to the basal levels. Increased fluorescence relative to the control indicated higher α-syn levels. For each plate, technical replicates were averaged to yield one value per condition; The mean values of the parallel replicates were presented as bar charts ±SD and statistical analysis was performed in GraphPad Prism 9 using a One-way ANOVA followed by Dunnett’s *post hoc* test, with significance set at *p* ≤ 0.05.

Images were generated 5 days after incubation of NL5901 nematodes with respective treatment (Ctr (+) and FE, Mt, Mq, and Sp at 200 μg/mL) for comparison with the untreated population (Ctr); then the worms were immobilized with 0.2% sodium azide. Images were acquired using a Zeiss Z1 Axio Observer inverted fluorescence microscope equipped with an AxioCam MRm camera and a GFP filter set (excitation 470/40 nm, emission 525/50 nm). For visualization, brightness and contrast were adjusted in ImageJ using linear settings that were applied identically across all images within the respective stack. Images are displayed in the green fluorescence channel.

#### 2.6.6 Heat shock resilience assay

NL5901 *C. elegans* were used in three parallel replicates. Culture preparation followed the same procedure as for the survival assay. Sterilized L3 larvae were incubated in triplicate wells with the test samples at the respective concentrations, a positive control (200 µM epigallocatechin gallate; (Ctr+)), or vehicle control (0.7% DMSO) for 4 days at 20 °C. Worms were then exposed to heat stress by shifting to 37 °C for 3.5 h, followed by recovery at 20 °C. Survival was assessed 24 h after heat stress as described for the survival assay. Data are presented as mean survival rate ±SD and plotted using GraphPad Prism 9 and statistical analysis was performed using a One-way ANOVA followed by Dunnett’s *post hoc* test, with significance set at *p* ≤ 0.05.

#### 2.6.7 β-Amyloid induced paralysis assay

Synchronized CL4176 10–30 L1 larvae were dispensed into the wells of a 24-well plate- filled with solid agar- and fed with OP50 *E. coli* (3 mg/mL). Larvae were treated with 200 μg/mL FE, vehicle control (ddH_2_O), or 6 mM caffeine (positive control) in triplicate wells. For each condition, an average of 60 worms was used (*n* ≈ 60, distributed to three wells in total). Plates were sealed with parafilm and incubated at 16 °C. Once worms reached ∼400 µm in length, plates were shifted to 25 °C (defined as time 0 h). After 48 h, the paralysis rate in each well was assessed under a dissecting microscope. Worms were scored as paralyzed when they displayed no body movement except for head bending and pharyngeal pumping. The mean paralysis rate of three replicate wells was plotted as bar chart ±SD and statistically analyzed using GraphPad Prism 9 and One-way ANOVA followed by Dunnett’s *post hoc* test, with significance defined as *p* ≤ 0.05.

### 2.7 Cell culture experiments

#### 2.7.1 Source and culturing of HMC3 cells

The human microglial cell line HMC3 (ATCC #CRL-3304, Lot-70046457) was cultured in DMEM/F-12 (Sigma #D8062) supplemented with 10% FCS (Sigma #F7524) and 1% penicillin/streptomycin (Sigma #A5955) at 37 °C and 5% CO_2_. Cells were regularly negative tested for *mycoplasma* contamination using MycoStrip™ - *Mycoplasma* Detection Kit (MycoStrip™ 100, InvivoGen).

#### 2.7.2 Stimulation of HMC3 cells with lipopolysaccharide (LPS)

For LPS stimulation, cells were seeded in 24-well plates at 7.5 × 10^4^ cells/well. After adherence overnight, cells were stimulated for 24 h with LPS from *E. coli* (100 ng/mL; Sigma #L5024, Lot-0000235347) and simultaneously treated with 1, 10, or 100 μg/mL of FE, Mq, Mt, Sp, or 5 µM dexamethasone as a positive control (Ctr (+)). DMSO (Honeywell, #D5879) was used as a solvent and added accordingly at 0.1% as control treatment (Ctr). Supernatants were then collected and cells harvested for RNA isolation.

#### 2.7.3 RNA isolation, cDNA synthesis and quantitative real-time PCR

RNA was isolated from the harvested cells after LPS-stimulation and treatment using the ReliaPrep RNA Cell MiniPrep System (Promega, #Z6010) following the manufacturer’s protocol. Briefly, cells were lysed in guanidine thiocyanate and 1-thioglycerol to inactivate RNases and disrupt nucleoprotein complexes. Lysates were applied to spin columns, treated with RNase-free DNase I to remove genomic DNA, washed, and eluted in nuclease-free water. First-strand cDNA was synthesized from 0.5 µg total RNA using iScript Reverse Transcription Supermix (BioRad, #172–5270) in a final reaction volume of 20 µL according to manufacturer’s instructions. Reverse transcription was performed at 25 °C for 5 min, followed by 42 °C for 30 min, and enzyme inactivation at 85 °C for 5 min. Multiplex quantitative PCR (qPCR) was performed using the Bio-Rad CFX Opus 96 system and a validated multiplex panel with probe-based detection. For each reaction, 50 ng cDNA template was combined with iQ™ Multiplex Powermix (Bio-Rad, #1725849), sequence-specific primers, and fluorescently labeled probes. The final reaction volume was 20 µL. The thermal cycling protocol consisted of an initial activation at 95 °C for 2 min, followed by 40 cycles of denaturation at 95 °C for 10 s and annealing/extension at 60 °C for 45 s. Fluorescence was measured in all channels. Expression of mRNA was evaluated using BioRad pretested primers and amplification was normalized to the reference gene *RPS18* (qHsaCEP0040177). Relative gene expression levels were determined by normalizing the Ct value of each target gene to the Ct value of the reference gene Rps18 (ΔCt = Ct_target - Ct_Rps18). Relative expression was calculated as 2^(-ΔCt). Primers tested in this experiment and purchased from BioRad were as follows: *IL1ß* (qHsaCIP0033362), *IL6* (qHsaCEP0051939) and *MCP-1* (CCL2; qHsaCID0011608).

#### 2.7.4 Cytokine secretion analysis *via* Bio-Plex assay

Cytokine levels in the supernatants of the LPS stimulation experiments were quantified using the Bio-Plex Pro™ Human Cytokine Panel (Bio-Rad, #171304090M), targeting MCP-1 (CCl2, #171B5021M). Assays were performed according to the manufacturer’s protocol, and fluorescence data were acquired using the Bio-Plex system.

#### 2.7.5 Annexin V apoptosis assay

For assaying apoptosis, HMC3 cells were seeded in 12-well plates at a density of 1 × 10^5^ cells/well and treated after overnight adherence with SP69, Mi, Mt, Sp (each at 1, 10, and 100 μg/mL), or 5 µM dexamethasone (Sigma, #D2915) for 24 h. DMSO (0.1%) served as vehicle control. Apoptosis was assessed using the APC Annexin V Apoptosis Detection Kit with PI (BioLegend, #640932) and analyzed by flow cytometry according to manufacturer’s instructions. FACS-analysis was performed at a NovoCyte flow cytometer and evaluated using the NovoExpress software.

#### 2.7.6 LDH cytotoxicity assay

Frozen supernatants from the LPS stimulation experiments were analyzed for cytotoxicity using the LDH Cytotoxicity Assay Kit (Abcam, #197004) in a 96-well format according to manufacturer’s instructions. Shortly, 10 µL of supernatants were incubated with LDH Reaction Mix (100 µL) for 30 min at room temperature. Absorbance was measured at 450 nm (reference 650 nm). Fluorescence was measured at Ex/Em = 535/587 nm. Cytotoxicity is calculated as the percentage of LDH released from test samples relative to total LDH release based on a cell lysis sample control.

#### 2.7.7 Data analysis and presentation

Data were analyzed using GraphPad Prism 10 (GraphPad Software, La Jolla, CA) and are means with individual values from two independent experiments ±SD (*n* = 6). Experimental groups were compared using One-way analysis of variance (ANOVA) followed by Dunnett post-tests vs. LPS control or Brown-Forsythe and Welch ANOVA tests followed by Dunnett T3 post-test vs. LPS control in case of unequal standard deviations, respectively.

## 3 Results

### 3.1 Phytochemical characterization of the hydroethanolic *F. ulmaria* extract (FE)

**FIGURE 1:**
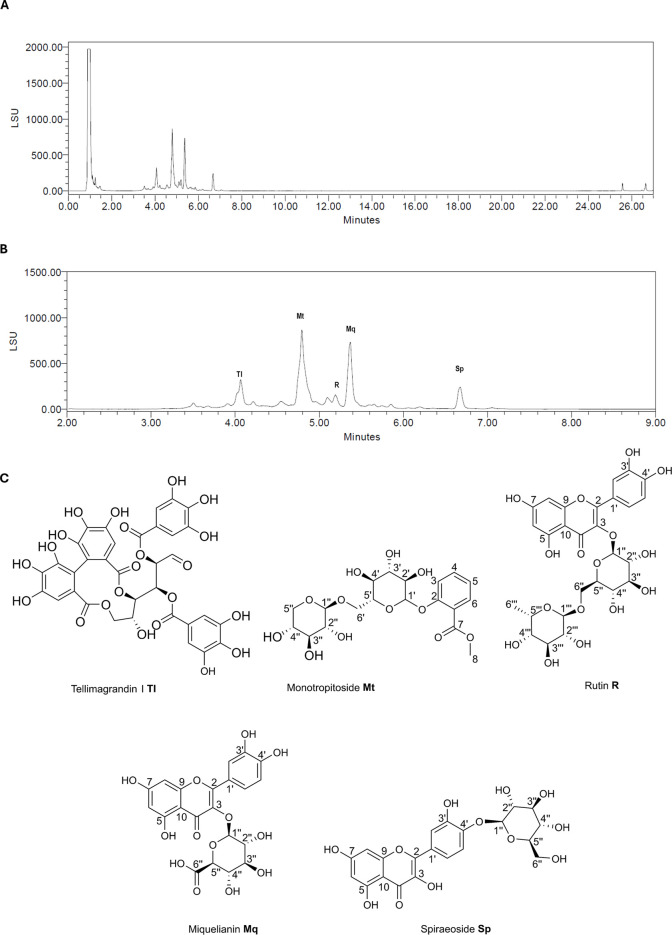
**(A)** UHPLC-ELSD chromatogram of FE. **(B)** Close up of A with annotated FE constituents. Their identity was confirmed by reference compounds. **(C)** Chemical structures of annotated constituents from FE.

The hydroethanolic extract (60% (m/m)) of the aerial parts of *F. ulmaria* (FE) was subjected to phytochemical analysis using a UHPLC system equipped with a photodiode array detector (PDA), evaporative light scattering detector (ELSD, Figure 1), and a single quadrupole mass detector (QDa). Comprehensive phytochemical profiling of FE was conducted using UHPLC-PDA-MS analysis in both positive and negative ionization modes. Notably, the ionization efficiency was markedly stronger in the negative mode. The metabolite spectrum was predominantly characterized by flavonoid glycosides and other phenolic constituents, including salicylic acid derivatives and hydrolysable tannins. The main constituents have been identified as tellimagrandin I (TI), monotropitoside (Mt), rutin (R), miquelianin (Mq), and spiraeoside (Sp) Figure 1 shows the ELSD chromatogram of FE along with the annotated constituents. Further details on the dereplication process are provided in Supplementary Table 1).

### 3.2 *In vivo* tolerability and health span promoting effects of FE in N2 nematodes

**FIGURE 2:**
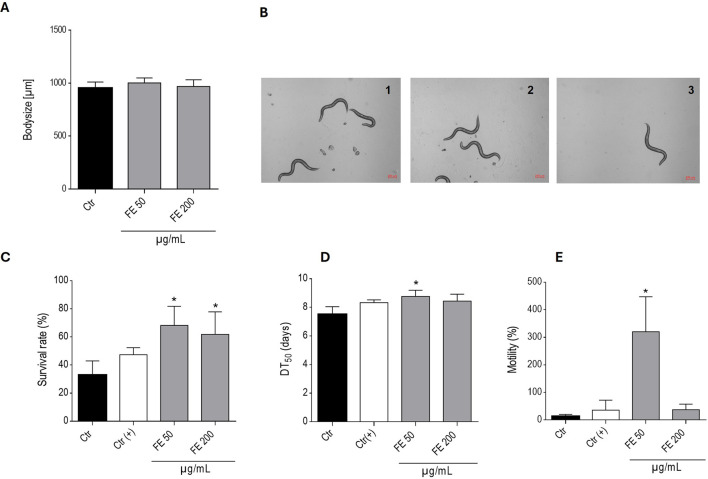
Effects of FE at given concentrations on the lifespan and healthspan of N2 wild-type *C. elegans*. **(A)** presents the mean body length of 21 adult N2 nematodes exposed to FE at respective concentrations ± SD for 5 days. **(B)** present exemplary pictures of adult N2 nematodes, treated with the vehicle control 0.7% DMSO (Ctr) (1), 50 μg/mL (2) and 200 μg/mL (3) FE after 5 days of incubation. Bars present **(C)** the mean survival rate (%) of N2 nematodes at the given conditions on the 11^th^ day of the experiment ± SD of three parallel experiments. 0.7% DMSO served as vehicle control (Ctr); 200 µM cyanidin chloride was used as positive control (Ctr(+)); **(D)** the mean DT_50_ of N2 worms in days ± SD of three parallel experiments. 0.7% DMSO served as vehicle control (Ctr); 200 µM cyanidin chloride was used as positive control (Ctr(+)); **(E)** the mean motility of N2 nematodes ± SD on the 7^th^ day of experiment, expressed as % of the basal activity. 0.7% DMSO served as vehicle control (Ctr); 200 µM cyanidin chloride was used as positive control (Ctr(+)). For **(A)** and **(C**–**E)** statistical significance was assessed by One-way ANOVA and Dunnett’s post-test (*p ≤ 0.05).

FE was evaluated for its effects on body size, lifespan, survival and motility to assess both tolerability and potential health-promoting properties in wild-type N2. As shown in Figures 2A–D, FE was well tolerated by the nematodes up to a concentration of 200 μg/mL. Treated worms showed normal development, body size and lifespan. Notably, treatment with 50 and 200 μg/mL FE doubled and significantly enhanced the survival rates by 88% respectively, whereas 50 μg/mL FE additionally significantly prolonged the lifespan of the nematodes, delaying the DT_50_ by 17%. In addition, semi-automated infrared-based motility tracking of worms on the 7^th^ day of adulthood revealed a significantly increased locomotor activity of worms exposed to 50 μg/mL FE (Figure 2E).

### 3.3 FE reduces α-synuclein-associated pathology in transgenic NL5901

**FIGURE 3:**
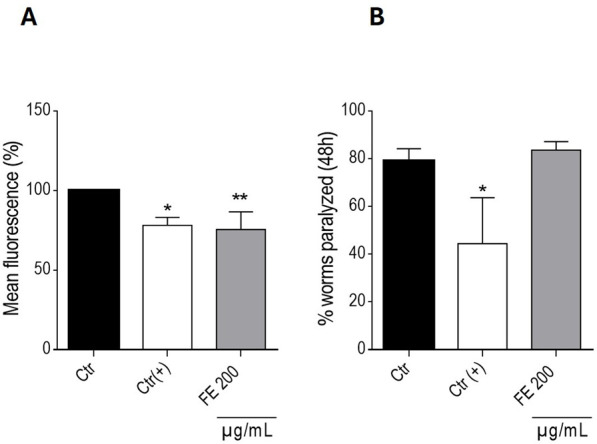
Effects of FE at given concentrations on **(A)** the mean YFP fluorescence (%) ± SD of NL5901 worms on the 5th day of experiment under the given conditions. 0.7% DMSO served as vehicle control (Ctr); 2.5 mM levodopa was used as positive control (Ctr(+)). **(B)** (%) paralyzed CL4176 worms, 48 h after Aβ-induction. 6 mM caffeine was used as positive control (Ctr(+)). Statistical significance was assessed by One-way ANOVA and Dunnett’s post-test (*p ≤ 0.05; **p ≤ 0.01).

Following the observation of extended lifespan and improved motility in FE treated wild-type *C. elegans*, the extract was evaluated for its neuroprotective activity in well-established transgenic models, directly targeting α-synuclein (α-syn) and β-amyloid (Aβ)-induced proteotoxicity (Figure 3). While 200 μg/mL FE did not affect acute Aβ-induced paralysis in the CL4176 *C. elegans* strain, it elicited a significant 24.5% reduction in the mean fluorescence of YFP-tagged α-syn in NL5901 nematodes. The following studies set out to explore the capacity of FE to mitigate α-syn-driven proteotoxicity, with the dual objective of (i) uncovering the bioactive metabolites of FE and (ii) defining their modulatory impact on neuroinflammatory processes.

### 3.4 Tracking the bioactive metabolites in FE

#### 3.4.1 Flash chromatographic fractionation of FE

To identify which metabolites contribute to the observed protective effect of FE towards α-syn proteotoxicity in NL5901, the multicomponent extract was fractionated by flash chromatography. This step yielded 19 MFs, containing both major and minor constituents. UHPLC-ELSD and UHPLC-MS analyses presented the efficient separation of the major constituents in FE (Supplementary Table 1) throughout the MFs and unveiled the remarkable diversity of minor ones, concealed in the unfractionated matrix. UHPLC- ELSD chromatograms of the MFs are presented in the supplementary information (Supplementary Figure 1).

#### 3.4.2 *In vivo* identification of bioactive MFs

**FIGURE 4:**
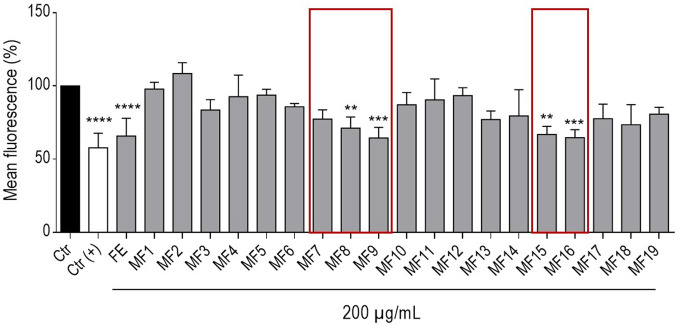
Bar chart presents the mean YFP fluorescence (%) ± SD of NL5901 worms treated with 200 μg/mL of FE and its MFs (MFx), on the 5^th^ day of experiment under the given conditions. 0.7% DMSO served as vehicle control (Ctr); 2.5 mM levodopa was used as positive control (Ctr(+)). Statistical significance was assessed by One-way ANOVA and Dunnett’s post-test (*p ≤ 0.05; **p ≤ 0.01; **p ≤ 0.001; **p ≤ 0.0001). The most bioactive MF packages (MF7-9 and MF15-16) are highlighted by red boxes.

Following fractionation of FE, all 19 MFs along with FE were tested at a concentration of 200 μg/mL for their impact on α-syn mediated proteotoxicity in transgenic NL5901 nematodes. As depicted in Figure 4, the results point toward the involvement of multiple constituents in mediating the reduction of mean YFP fluorescence, with pronounced activities observed across flavonoid glycosides enriched MFs (MF7-16). Among these, MF8 and MF9, as well as MF15 and MF16, exhibited the most significant effects, reducing the mean YFP-fluorescence by 29%, 35.5%, 33% and 35% (*p* ≤ 0.01; *p* ≤ 0.001; *p* ≤ 0.01; *p* ≤ 0.001), respectively.

#### 3.4.3 Correlation of chemical profiles and bioactivities

**FIGURE 5:**
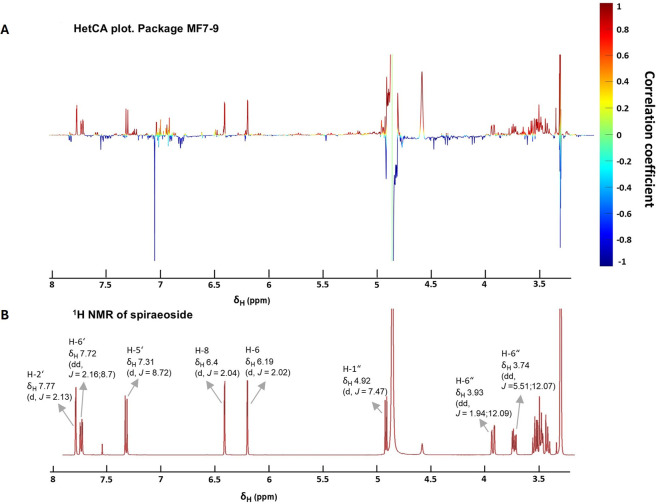
^1^H NMR-based biochemometric identification of the active constituent of FE, responsible for the decreased α-syn::YFP levels in transgenic NL5901 *C. elegans*. **(A)** HetCA plot comprising the ^1^H NMR data of MF7-9. The color code is based on the correlation coefficient, whereby red signals are positively and blue signals are negatively correlated with bioactivity. **(B)** ^1^H NMR spectrum of the reference Sp, measured in deuterated methanol.

**FIGURE 6:**
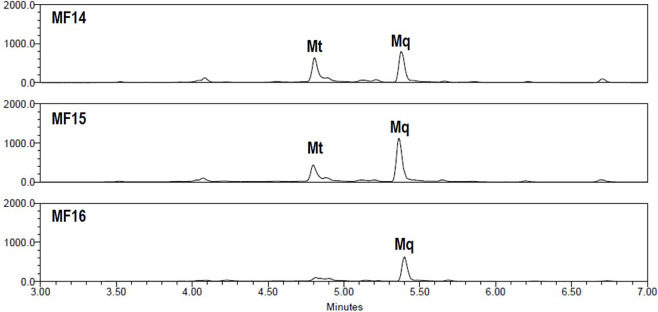
ELSD chromatograms of FE-derived MF14-MF16 presenting the ratios of the major constituents monotropitoside (Mt) and miquelianin (Mq).

Investigating the chemical profiles of MF8-9 and MF15-16 (Supplementary Figure 1) revealed large differences in their chemical complexity, which markedly influenced the approach used to identify the bioactive compounds. In the case of MF7-9, both MS spectra and ELSD chromatograms showed a highly complex and heterogeneous composition, characterized by numerous minor constituents alongside the major compounds TI in MF8 and Sp in MF9. The abundance of metabolites made a direct manual assignment of the bioactive compounds difficult and necessitated the use of advanced tools to identify the active secondary metabolites in our proteotoxicity model. Thus, we integrated the biochemometric ^1^H NMR approach (ELINA) into this study (Grienke et al., 2019). The analysis identified signals corresponding to the flavonoid glycoside Sp as positively correlated with the reduction in YFP fluorescence (Figure 5A). The identity of the proposed bioactive compound was confirmed by aligning the ^1^H NMR spectrum of Sp from an authentic standard with the respective signals in the HetCA plot (Aligiannis et al., 2016) (Figure 5B). The ELSD chromatograms of MF15-16 showed a significantly simpler composition, allowing the straightforward prediction of Mq as active constituent and Mt as inactive one (Figure 6).

### 3.5 Impact of Mt, Sp, and Mq on α-syn proteotoxicity in *C. elegans*

**FIGURE 7:**
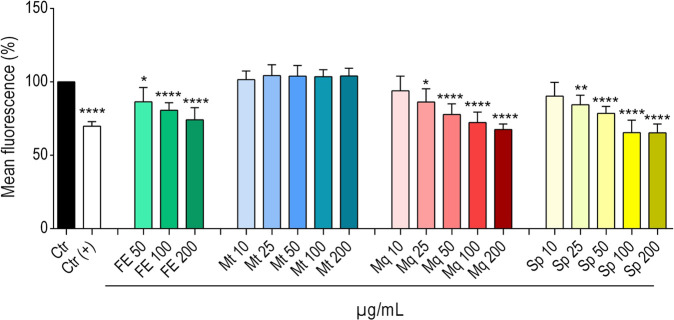
Bars represent the mean YFP fluorescence (%) ± SD of NL5901 worms after the treatment with FE and the natural compounds monotropitoside (Mt), miquelianin (Mq) and spiraeoside (Sp) at the given concentrations. 0.7% DMSO served as vehicle control (Ctr); 2.5 mM levodopa was used as positive control (Ctr(+)). Statistical significance was assessed by One-way ANOVA and Dunnett’s post-test (*p ≤ 0.05; **p ≤ 0.01; ****p ≤ 0.0001).

Based on the correlation experiments performed, the flavonoid glycosides Sp and Mq were classified as active, while Mt was predicted to be inactive or negatively correlated with the observed bioactivity. To validate these predictions, all three compounds were subjected to concentration-dependent evaluation (10–200 μg/mL) in the NL5901 *C. elegans* α-syn proteotoxicity model (Figure 7). A significant dose-dependent reduction in mean YFP fluorescence was observed upon treatment with Sp and Mq at concentrations ≥25 μg/mL, confirming their bioactivity. Mq led to reductions of 6%–32%; similarly, Sp decreased the fluorescence intensity between 10% and 35% with increasing concentrations. In contrast to Mq and Sp, Mt did not exert any significant changes in fluorescence at any tested concentration. Representative pictures of the worms are provided in Supplementary Figure 3.

### 3.6 Structural relationships

**FIGURE 8:**
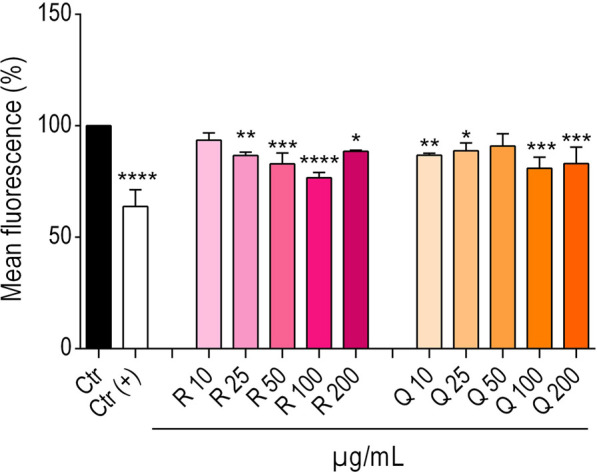
Bars represent the mean YFP fluorescence (%) ± SD of NL5901 worms after the treatment the FE-derived natural compounds rutin (R) and quercetin (Q) at the given concentrations. 0.7% DMSO served as vehicle control (Ctr); 2.5 mM levodopa was used as positive control (Ctr(+)). Statistical significance was assessed by One-way ANOVA and Dunnett’s post-test (*p ≤ 0.05; **p ≤ 0.01; ***p ≤ 0.001 ****p ≤ 0.0001).

The structural comparison of Mq (quercetin-3-O-glucuronide) and Sp (quercetin-4′-O-glucoside) (Figure 1C) raised the question to which extent the aglycon scaffold Q and the sugar moiety contribute to the modulation of α-syn::YFP fluorescence observed in NL5901. To tackle this question, Q and the flavonoid glycoside quercetin-3-O-glucorhamnoside (R), detected in low quantities in MF11-16 (Supplementary Table 1; Supplementary Figure 1) were subjected to bioactivity assessment under identical assay conditions (10–200 μg/mL; Figure 8). Both Q and R led to moderate (not dose-dependent) reductions in YFP fluorescence between 9% and 19%, and 6% and 23%, respectively.

### 3.7 Sp and Mq improve thermotolerance and survival in NL5901

**FIGURE 9:**
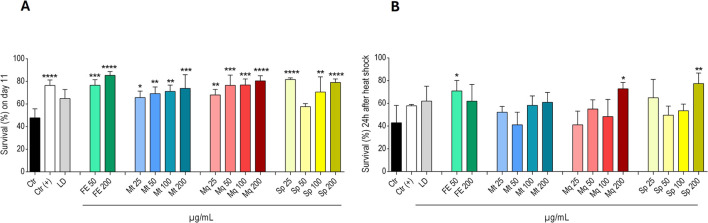
Effect of FE and derived natural compounds monotropitoside (Mt), miquelianin (Mq) and spiraeoside (Sp) at the given concentrations and 2.5 mM levodopa (LD) on **(A)** the mean survival rate (%) ± SD of NL5901 nematodes on the 11^th^ day of adulthood. **(B)** the mean survival rate (%) ± SD of NL5901 worms, incubated with the samples for four days, 24h after being heat shocked at 37.0 °C for 3.5 hours. For **(A)** and **(B)**: 0.7% DMSO served as vehicle control (Ctr); 200 μM epigallocatechingallate was used as a positive control (Ctr(+)). Statistical significance was assessed by One-way ANOVA and Dunnett’s post-test (*p ≤ 0.05; **p ≤ 0.01; ***p ≤ 0.001 ****p ≤ 0.0001).

The effects of FE and its derived constituents Sp, Mq, and Mt were assessed for their ability to improve survival and mitigate heat-induced stress in worms. This approach enabled assessment of whether the observed effects extend beyond α-syn mediated proteotoxicity to broader stress- and age-related signaling pathways. The survival assay revealed survival promoting effects for all tested samples, with significant survival rates observed for Mt and Mq at 25–200 μg/mL (Mt: +37%, +45%, +49%, +55%; Mq: +42%, +60%, +61% and +68% vs. vehicle control, respectively). Sp enhanced survival at 25 and 100–200 μg/mL (+71%, +48% and +66% vs. vehicle control), and the crude extract enhanced the survival at 50 and 200 μg/mL by 60% and 79% compared to the vehicle control (Figure 9A). Additionally, nematodes treated with FE (50 and 200 μg/mL) and Sp, Mq and Mt at concentrations between 25 and 200 μg/mL tolerated heat-induced stress better than the vehicle control (Figure 9B). Remarkably, 200 μg/mL Mq and Sp significantly surpassed the control’s survival by 70% and 80% (p ≤ 0.05). The extract FE enhanced thermotolerance in the worms at both concentrations by 65% and 44%. Mt exhibited milder but noticeable effects at 100 and 200 μg/mL (+36% and +42% vs. vehicle control, respectively), although these did not reach statistical significance and lower concentrations did not influence the heat resistance of the nematodes.

### 3.8 Sp and Mq suppress pro-inflammatory gene expression in microglial-like cells

**FIGURE 10:**
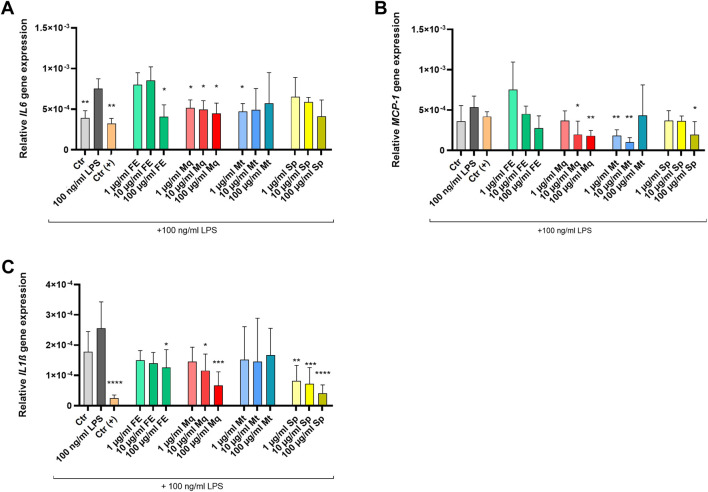
Effects of FE and its constituents on LPS-induced cytokine expression in HMC3 cells. **(A–C)** LPS (100 ng/ml) increased IL-6, CCL2 (MCP-1), and IL-1β mRNA levels. 5 μM Dexamethasone served as a positive control (Ctr (+)), 0.1% DMSO served as vehicle control (Ctr). Bars present means ± SD and individual values from two independent experiments (n=6); *p ≤ 0.05, **p ≤ 0.01, ***p ≤ 0.001, ****p < 0.0001 compared to LPS. Experimental groups were compared using One-way analysis of variance (ANOVA) followed by Dunnett post-tests vs. LPS control or Brown-Forsythe and Welch ANOVA tests followed by Dunnett T3 post-test vs. LPS control in case of unequal standard deviations, respectively.

To evaluate whether FE and its constituents also exert anti-inflammatory effects, we investigated their impact on pro-inflammatory cytokine expression in the human microglial clone 3 (HMC3) cell line stimulated by LPS. Thereby, LPS-stimulated HMC3 cells showed significantly increased mRNA levels of *IL-6* (Figure 10A), *MCP-1* (*CCL2*) (Figure 10B), and *IL-1β* (Figure 10C), which confirmed the activation of the inflammatory response in the experimental setting.

Treatment with FE (100 μg/mL) and its isolates Mq (1, 10, 100 μg/mL) and Mt (1 μg/mL) significantly reduced *IL-6* expression. Among the tested isolates, Sp showed a concentration-dependent trend toward *IL-6* reduction, though the effect did not reach statistical significance. *MCP-1* expression was attenuated by FE and all tested compounds. Significant reductions were observed with Mq (10 and 100 μg/mL), Mt (1 and 10 μg/mL), and Sp (100 μg/mL). *IL-1β* levels were significantly decreased following treatment with FE (100 μg/mL), Mq (10 and 100 μg/mL), and Sp (1–100 μg/mL). The positive control dexamethasone (5 µM) markedly suppressed *IL-6* and *IL-1β* gene expression.

**FIGURE 11:**
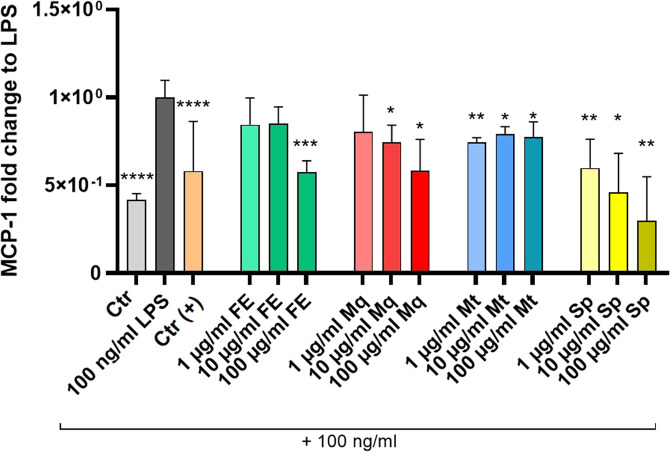
MCP-1 protein levels in LPS-stimulated HMC3 cells after treatment with FE and its constituents. LPS (100 ng/mL) markedly increased MCP-1 protein expression. 5 μM Dexamethasone served as a positive control (Ctr (+)), 0.1% DMSO served as vehicle control (Ctr). Bar charts present means ± SD and individual values from two independent experiments (n=6); *p ≤ 0.05, **p ≤ 0.01, ***p ≤ 0.001, ****p < 0.0001 compared to LPS. Experimental groups were compared using One-way analysis of variance (ANOVA) followed by Dunnett post-tests vs. LPS control or Brown-Forsythe and Welch ANOVA tests followed by Dunnett T3 post-test vs. LPS control in case of unequal standard deviations, respectively.

These anti-inflammatory effects were further investigated at the protein level using MCP-1 as an example. Treatment with FE (100 μg/mL), Mq (10 and 100 μg/mL), Mt (1–100 μg/mL), and Sp at all tested concentrations significantly reduced MCP-1 levels (Figure 11). With the exception of Mt, treatment effects were concentration-dependent with more pronounced inhibitory effects at the highest concentration of FE, Mq and Sp.

## 4 Discussion

### 4.1 FE modulates health span and α-syn levels in *C. elegans*

This study investigated FE, a hydroethanolic extract from the aerial parts of *F. ulmaria* with, known anti-inflammatory activity, for its ability to interfere with mechanisms relevant to aging and neurodegeneration using the model organism *C. elegans* and human microglia.

The pathology of human PD is strongly associated with aging which impairs among others the proteostasis network (Hipp et al., 2019) and diminishes the cells’ ability to manage misfolded proteins like α-syn. This cellular defense system includes the heat shock response (HSR) (Hsu et al., 2003; Kirchweger et al., 2023; Morley and Morimoto, 2004). It counteracts protein misfolding (Labbadia and Morimoto, 2015) by directing chaperones and heat shock proteins (HSPs) *via* heat shock factor-1 (HSF-1) to refold or degrade misfolded proteins (Aridon et al., 2011). With aging, HSF-1 activity declines, which exacerbates protein aggregation and triggers chronic microglial activation, leading to inflammation (Gomez-Pastor et al., 2018; Zhang et al., 2005; Thakur and Nehru, 2014). In human PD, reducing α-syn levels is considered a promising therapeutic strategy in PD, targeting the key drivers of neuroinflammation and neurotoxicity (Morris et al., 2024). Accordingly, the suppression of protein accumulation through reduced gene expression, as well as the reduction of aggregation or promotion of depolymerization and degradation, are potential strategies to slow down or prevent disease progression (Dehay et al., 2015).

As a multicellular organism with a high degree of conserved aging mechanisms, *C. elegans* can mirror the responses of the proteostasis network to α-syn. Previous work in *C. elegans* has shown that longevity pathways including reduced insulin/IGF-1 signaling (IIS) enhance proteostasis through increased entry of transcription factors including abnormal dauer formation protein 16 (DAF-16; ortholog of mammalian FOXO: Forkhead-Box O), skinhead-1 (SKN-1; ortholog of mammalian NRF2: Nuclear Factor-erythroid 2-Related Factor 2), and HSF-1. In the nucleus they activate chaperones and other protective proteins to combat oxidative stress (Hipp et al., 2019).

In addition, both, the conserved MPK-1 (homolog to mammalian ERK) mitogen-activated protein kinase (ERK-MAPK) and p38-MAPK cascade contribute to longevity and HSR in *C. elegans* by phosphorylating SKN-1, which allow its nuclear accumulation (Okuyama et al., 2010). Moreover PMK-1 (ortholog to human MAPK-11 and MAPK-14) promotes HSF-1-dependent HSR in *C. elegans*. Together with mammalian data showing direct phosphorylation of HSF-1 by p38, PMK-1 may directly activate HSF-1 in worms (Mertenskötter et al., 2013). A direct mechanistic link between MAPK signaling and α-syn accumulation in *C. elegans* has not yet been demonstrated. However, recent studies provide correlative evidence suggesting that MAPK pathways, particularly PMK-1/p38 and MPK-1/ERK, may influence α-syn-associated phenotypes in NL5901 (Huang et al., 2024).

Of note, research on transgenic NL5901 nematodes has demonstrated that longevity-associated genes, such as the silent information regulator 2 homolog 1 (*sir-2.1)* an upstream regulator of DAF-16 and HSR components, can affect the formations of α-syn inclusions (van Ham et al., 2008).

The transgenic *C. elegans* strain NL5901 expresses YFP-tagged human α-syn in its body wall muscles, where it forms visible inclusions over time. Because NL5901 expresses α-syn exclusively in the muscle cells and lacks a complex CNS, it does not allow direct assessment of dopaminergic decline or blood-brain-barrier (BBB) permeability. Nevertheless, assessing the α-syn::YFP fluorescence offers initial insights into the modulation of the α-syn levels (referred to as accumulation in this work) which may reflect reduced expression, improved degradation or clearance.

The benefits of FE on survival and motility in *C. elegans* suggest the modulation of age-associated pathways that delay aging (Son et al., 2019; Rollins et al., 2017) and counteract the age-associated loss of muscle integrity (Herndon et al., 2002). Preserving mobility at advanced age not only indicates improved health span in worms (Rollins et al., 2017), but may translate into notable gains in quality of life of PD patients, who experience motor impairments from progressive muscle loss and functional decline.

Expanding our research toward key proteins of neurodegeneration, we focused on two proteins central to neurodegenerative pathology: Aβ and α-syn, using two transgenic *C. elegans* strains CL4176 and NL5901. Interestingly, FE selectively and dose-dependently reduced α-syn::YFP fluorescence in NL5901 worms with pronounced reduction observed at 200 μg/mL.

In this context, reduced α-syn-levels alongside increased thermotolerance and survival in FE-treated NL5901 worms may reflect HSF-1 mediated enhancement of proteostasis and longevity. Although a generalized HSR would likely affect both Aβ and α-syn, FE selectively reduced α-syn::YFP levels without altering Aβ-induced proteotoxicity in CL4176 worms. This specificity suggests additional mechanisms beyond general stress response pathways that may target α-syn directly. Our study is the first to demonstrate the life- and health-promoting effects of FE in *C. elegans* and to provide initial indication of α-syn-specific reduction of protein levels. From a translational perspective, accumulating α-syn forms toxic oligomers that propagate between cells, impair synaptic electrophysiology, and activate microglia, driving them into a pro-inflammatory state *via* nuclear factor kappa-light-chain-enhancer of activated B cells (NF-κB) mediated signaling (Gao et al., 2023).

In LPS-stimulated human microglia, FE significantly reduced the expression of pro-inflammatory cytokines *IL-1β* and *IL-6*, at 100 μg/mL, and reduced the *MCP-1* expression at 100 μg/mL encoding a chemoattractant protein, implicated in the infiltration of monocytes into the brain (Gao et al., 2023). These findings extend the known anti-inflammatory properties of *F. ulmaria* (Katanić et al., 2018; Samardžić et al., 2016; Arsenijevic et al., 2021) to a neurological context, highlighting FE’s multifaceted activity spectrum.

Together, results from this combined *in vivo*-*in vitro* approach demonstrate FE’s potential to mitigate neurodegenerative processes through anti-inflammatory action and protection against α-syn proteotoxicity. Considering the chemical complexity of FE, our investigations aimed to identify those constituents with geroprotective activity observed in *C. elegans* and to assess their ability to counteract neuroinflammation *in vitro.*

### 4.2 Tracking the bioactive metabolites in FE

Overcoming the chemical complexity in bioactive crude extracts is a major challenge in the discovery and investigation of natural products. Fractionation of an extract is a suitable process to simplify the metabolites’ complexity towards isolation of the main constituents, while at the same it enables to enrich minor metabolites in individual fractions. Analysis of the chemical profiles of MF8-9 and MF15-16 (Supplementary Figure 1) revealed substantial differences in complexity. This divergence in complexity guided the choice of analytical strategies to identify the bioactive constituents. In particular, MF8-9 required advanced tools to address the extensive structural diversity present in these fractions.

The ^1^H NMR-based biochemometric approach ELINA (**El**iciting **N**ature’s **A**ctivities) facilitates the early identification of active constituents from complex mixtures, before purification (Grienke et al., 2019). It is a resource-efficient strategy, since it not only reduces the probability of re-isolating known compounds but also captures minor constituents that may contribute to bioactivity (Grienke et al., 2019; Zwirchmayr et al., 2023). The method relies on correlating bioactivity data with proton signals from ^1^H NMR spectra of complex mixtures through heterocovariance (HetCA) analysis (Aligiannis et al., 2016) which allows for the classification of signals as positively and negatively correlated to the observed bioactivity. Central to this approach is the gradual progression of activity observed in a consecutive series of at least three MFs (referred to as “packages”) and their respective varying constituent quantities, which are reflected by the signal intensity in the ^1^H NMR spectra (Grienke et al., 2019; Zwirchmayr et al., 2023; Wasilewicz et al., 2024).

Due to the gradual increase in activity (i.e., decrease of fluorescence intensity in transgenic NL5901 nematodes) from MF7 to MF9 and their slightly overlapping chemical profile, this MF package fulfilled the criteria for HetCA analysis. For this purpose, NMR samples of the individual MF7-9 were prepared in deuterated methanol under identical conditions and subjected to ^1^H NMR spectroscopy using uniform acquisition parameters to ensure comparable signal-to-noise ratios.

The chemical composition of MF14-16 was less complex than MF7-9 (Figure 6; Supplementary Figure 1). Their ELSD chromatograms indicated that these MFs were predominantly composed of the salicylic acid glycoside Mt and the flavonoid glucoside Mq, present in significantly varying ratios. Based on the chemical profiles of MF14-16, Mt was presumed to be pharmacologically less active or play a potential antagonistic role, while Mq was postulated as the main bioactive compound within these MFs.

### 4.3 Sp and Mq as dual modulators of α-syn pathology and neuroinflammation

#### 4.3.1 Sp and Mq mitigate α-syn pathology and thermotolerance in *C. elegans*

Through analytical and phytochemical analyses, the decreased α-syn::YFP fluorescence observed for FE could be attributed to the presence of Sp and Mq, whereas the abundant phenol glycoside Mt showed no activity on α-syn fluorescence in NL5901 nematodes.

Notably, flavonoid glycosides show increasing potential in preclinical neurodegenerative models by attenuating oxidative stress, reducing (neuro)inflammation and microglial activation, and supporting proteostasis (Chen et al., 2022). This compound class is pharmacologically versatile, acting on conserved aging- and stress-related pathways and targets, including IIS, NRF-2 and AMP-activated protein kinase (AMPK) (Rębas, 2025).

As typical for flavonoid glycosides, Sp (Nile et al., 2021; Liu et al., 2020; Wang et al., 2024) and Mq (Verjee et al., 2018) have documented *in vitro* anti-inflammatory and stress-modulatory activities relevant to neurodegeneration: Sp has been shown to activate the PI3K/Akt/NRF2 axis and suppress oxidative stress in cardiomyocytes (Liu et al., 2020), while Mq exerts anti-inflammatory effects by activating NRF2 in LPS-stimulated immune models (Yu et al., 2024) and modulating MAPK cascades (Ha et al., 2021). Although these mechanisms closely align with pathways known to modulate stress resilience and proteostasis in *C. elegans*, this study is the first to demonstrate a protective effect of Mq and Sp against α-syn accumulation in a living organism.

Notably, 10–200 μg/mL Sp and Mq reduced α-syn fluorescence in a clear dose-dependent manner, peaking at 200 μg/mL. The effects were accompanied by increased thermotolerance of NL5901 worms treated with compounds at 200 μg/mL.

In *C. elegans*, flavonoids promote longevity and stress resistance through conserved pathways and targets including IIS, AMPK, and the HSR axis. Although these activities have not yet been investigated specifically for Sp or Mq in *C. elegans*, their involvement is plausible and supported by findings of their aglycone Q (Ayuda-Durán et al., 2019; Sugawara and Sakamoto, 2020), offering a coherent mechanistic explanation for the observed lifespan extension, reduced α-syn accumulation, and increased thermotolerance.

Nevertheless, the effect on thermotolerance declined markedly at lower concentrations, yielding only modest survival benefits. This suggests that only higher doses are sufficient to elicit a robust HSR under severe heat stress. Considering the known correlation between reduced α-syn::YFP fluorescence and HSF-1 mediated thermotolerance (Govindan et al., 2018; Qu et al., 2022), our results indicate that the improved HSR likely contributes to the reduced α-syn accumulation observed in NL5901.

Interestingly, Mt slightly improved thermotolerance at 200, 100 and 25 μg/mL, accompanied by a dose-dependent survival increase. This enhanced HSR parallels previous evidence for salicylic acid- induced thermotolerance in plants (Zhang G. et al., 2023; Sangwan et al., 2022). To our knowledge, this is the first evidence of Mt on promoting survival and HSR in *C. elegans*. Although Mt has been described for its inhibitory activity against COX-2 (Michel, 2025) being a classical anti-inflammatory salicylate mechanism in mammals, it cannot explain the effects we observed in our study, because *C. elegans* lacks canonical COX orthologs.

Instead, Mt is supposed to act through evolutionary conserved stress and longevity pathways. Similar to other salicylates (e.g., acetylsalicylic acid and salicylic acid), which extend lifespan and improve stress resilience in *C. elegans* partly through IIS modulation (Wan et al., 2013; Shamalnasab et al., 2018), Mt may engage these mechanisms consistent with its observed thermotolerance and survival benefits.

Furthermore, recent findings indicate that salicylic acid derivatives can activate AMPK in *C. elegans*, promoting lifespan extension and mitochondrial stress resilience (Shamalnasab et al., 2018). In mammalian systems, salicylates exert anti-inflammatory effects not only through COX inhibition but also by altering MAPK and suppressing NF-κB signaling (Vittimberga et al., 1999). Since NF-κB is absent in *C. elegans* (Wang et al., 2006), this pathway cannot contribute mechanistically. Conversely, MAPK pathways are conserved in *C. elegans* and form a central part of its stress response machinery that indirectly controls proteostasis (Yuan et al., 2023; Uno and Nishida, 2016). Although Mt’s activity on MAPK signaling remains untested, it may contribute to the improved thermotolerance and survival of NL5901 nematodes.

Intriguingly, in contrast to the marked reductions seen with the tested flavonoid glycosides Mq and Sp, the herein observed effects of Mt occurred without a concomitant reduction in α-syn levels. Previously, sodium salicylate has been shown to activate HSR in rats by increasing HSF-1, HSP-27 and HSP-40 expression, whereas the levels of HSP-70 remained unchanged (Thakur and Nehru, 2014). HSP-70 is known to reduce α-syn aggregation *in vitro* (Roodveldt et al., 2009) and supports the detoxification of misfolded α-syn monomers/oligomers in *C. elegans* and *Drosophila melanogaster.* This lack of HSP-70 induction may therefore account for the improved thermotolerance without a concomitant reduction in α-syn levels.

The impact of both flavonoids Sp and Mq on HSP-70 expression remains to be investigated. Given their Q-based scaffold, an interaction with HSP-70 is plausible (Sugawara and Sakamoto, 2020; Ayuda-Durán et al., 2019), however, targeting this hypothesis was beyond the scope of the present study. In line with our findings, Mt has not been reported to modulate α-syn directly, whereas salicylic acid has been reported to slightly promote α-syn fibrillation *in vitro* (Ardah et al., 2014). In comparison, Mq was recently shown to moderately interfere with α-syn fibril formation *in vitro* (So et al., 2024). To our knowledge, so far there is no study that assesses the impact of Sp on α-syn aggregation; only the aglycone Q has been described to inhibit α-syn fibrillization *in vitro* (Zhu et al., 2013) and reduce aggregation in the NL5901 strain (Das et al., 2022).

In the light of previous reports, the role of the Q scaffold was examined more closely as potential contributor to the observed reductions in α-syn::YFP fluorescence upon treatment with Mq an Sp. Subsequent investigations confirmed the role for Q in the observed effects, though it appeared to be only a moderate factor. As reported previously (Day et al., 2003; Ader et al., 2001), differences in absorption and bioavailability mechanisms may also modulate the observed activities.

#### 4.3.2 Sp and Mq suppress pro-inflammatory gene expression in microglial-like cells

In line with previous reports describing the anti-inflammatory potential of *F. ulmaria* (Katanić et al., 2016) and its phenolic constituents, FE as well as Mq, Sp and Mt displayed significant anti-inflammatory effects in LPS-stimulated HMC3 microglia. They reduced *IL-6*, *MCP-1* (*CCL2*), and *IL-1β* expression and suppressed MCP-1 protein levels, showing magnitudes of inhibition comparable to the reference drug dexamethasone. For Mq and Sp, *IL-1β* and *IL-6* expression decreased with increasing concentrations, reaching the strongest inhibition at 100 μg/mL. Interestingly, *MCP-1* expression, reaching significance at 10 and 100 μg/mL, declined in a dose-dependent manner in response to Mq whereas for Sp, a significant reduction was observed only at the highest concentration (100 μg/mL). In contrast, Mt showed an inverse dose-response relationship for *IL-6* expression, with 1 μg/mL exerting the strongest inhibitory effect. Consistent with this pattern, the *MCP-1* expression was also reduced at 1 and 10 μg/mL Mt, with 10 μg/mL producing the stronger effect.

Although mRNA induction by LPS was not statistically significant after 24 h, this probably reflects delayed transcription dynamics. Changes at protein level, here exemplified by MCP-1, were significantly detectable, indicating effective cytokine translation and secretion at this time point.

To ensure that the anti-inflammatory effects did not result from impaired cell viability, we investigated the cytotoxicity of FE and its components in an LDH and Annexin V/PI apoptosis assay, and observed no cytotoxic or proapoptotic effects (Supplementary Figure 2).

Mt has been recently described as a natural salicylate derivative with NSAID-like activity that may confer neuroprotection through COX inhibition and anti-apoptotic mechanisms, while avoiding gastrointestinal side effects (Michel, 2025). Given its structural similarity to salicylic acid, an additional modulatory effect of Mt on NF-κB signaling is conceivable. To the best of our knowledge, this is the first report describing the effects of Mt on pro-inflammatory cytokine expression in human microglia, highlighting its effects on neuroinflammation.

To date, no direct evidence has demonstrated a neuroprotective effect of Sp; however, its potential has been suspected from its close structural similarity to flavonoids such as Mq, which have been shown to exert neuroprotective potential (Wang et al., 2024; Verjee et al., 2018). Our results provide the first experimental evidence that Sp exerts anti-inflammatory and potentially neuroprotective effects in microglial cells, identifying it along with Mq as a likely major contributor to FE’s observed activity in microglia.

### 4.4 Limitations and future perspectives

Overall, the effects observed for FE and its constituents Sp and Mq *in vitro* and *in vivo* complement previous *in vitro* data and highlight the therapeutic potential of these plant metabolites in modulating key pathways involved in neurodegenerative disorders like PD. In NL5901 *C. elegans*, α-syn is expressed under the *unc-54* promoter in body-wall muscle cells rather than in neurons (Bodhicharla et al., 2012). This disparity limits the direct modeling of neuronal vulnerabilities and synaptic dysfunction. Moreover, while total YFP fluorescence provides a sensitive, high-throughput readout of α-syn levels, it does not assess accumulation or aggregation dynamics, and the relative contribution of protein expression, biophysical inhibition of aggregation, or HSR-associated proteostasis remains unclear. Future mechanistic studies should employ aggregate-specific assays and gene-expression analyses to delineate these effects. Furthermore, it is important to acknowledge anatomical limitations of the worm model, namely the absence of a complex CNS and a BBB. As a result, compound uptake and distribution in *C. elegans* do not reflect mammalian pharmacokinetics, and the BBB cannot be addressed within this system. In this regard, Mq has been reported in a Caco-2 cell model to cross from the intestine to the central nervous system to a certain extent (Juergenliemk et al., 2003), whereas Sp has not yet been investigated in this regard. Future investigations should therefore examine the pharmacokinetic behavior, metabolism, and BBB permeability of Sp and Mq to assess translational relevance.

## 5 Conclusion

In summary, the complex extract FE and selected components thereof exhibit a multimodal therapeutic potential against key mechanisms underpinning neurodegeneration, particularly PD. FE itself significantly attenuates microglial inflammatory markers, enhances lifespan and health span and reduces α-syn levels in transgenic *C. elegans*, collectively indicating its geroprotective function and its capacity to interfere with PD from the neuroinflammatory and α-syn angle.

Among the major constituents of FE, Mt operates primarily as an anti-inflammatory agent, effectively suppressing MCP-1 in microglia, but does not directly impact α-syn levels in NL5901. In contrast, Mq and Sp exhibit a broader functional spectrum: both enhance thermoresistance, improve survival and decrease α-syn levels in *C. elegans*, presumably through proteostatic modulation. Sp and Mq further combine these effects with anti-inflammatory activity, indicating complementary actions in the field of PD. Importantly, their effects differ from those of other flavonoids in FE, and is not solely dependent on the Q backbone. Rather, specific substitution patterns and sugar moieties shape their neuroprotective properties.

Taken together, FE and specifically Mq and Sp emerge as promising candidates for the prevention or treatment of neurodegenerative diseases. Their capacity to converge on neuroinflammation, proteostatic balance, and aging-related signaling offers a compelling therapeutic profile. Future studies should elucidate the molecular mechanisms governing α-syn aggregate dynamics and assess the translational potential of these findings in mammalian models and ultimately in humans.

## Data Availability

The original contributions presented in the study are included in the article/Supplementary Material, further inquiries can be directed to the corresponding author.
