# Supplementary material for: Miquelianin and spiraeoside from Filipendula ulmaria mitigate α-synuclein accumulation in C.elegans and reduce the expression of neuroinflammatory cytokines in human microglia
Source: Front Pharmacol. 2026 Feb 11;16:1720314. doi: 10.3389/fphar.2025.1720314 (PMC12933199; doi:10.3389/fphar.2025.1720314)
Supplement: Supplementary file 1 [file Supplementaryfile1.pdf]

Supplementary Material

1 Supplementary Figures and Tables

1.1 Supplementary Tables

Supplementary Table 1. Analytical characteristics of constituents detected in the hydroethanolic *F. ulmaria* extract FE by UHPLC-PDA-MS and UHPLC-PDA-ELSD (corresponding to **Figure 1C**).

Compound ID	Annotation	MW [g/mol]	Negative mode [m/z]	Positive mode [m/z]	Absorption UV [nm]	Rt [min]
**Main constituents (as shown in the UPLC-ELSD chromatogram, Figure 1)**						
**TI**	telimagrandin I	786.6	Fragment ions 341.06; 392.5; 371.03	-	216.3;274	4.06
**Mt**	monotropitoside	446.4	481.02 $[M+Cl]^-$ 491.03 $[M+FA-H]^-$	469.03 $[M+Na]^+$	278	4.79
**R**	rutin	610.5	609.34 $[M-H]^-$	632.27 $[M+Na]^+$	253; 364.7	5.18
**Mq**	miquelianin	478.4	477.21 $[M-H]^-$	479.07 $[M+H]^+$ 501.09 $[M+Na]^+$	255.4;352.7	5.37
**Sp**	spiraeoside	464.4	463.2 $[M-H]^-$	465.12 $[M+H]^+$	255; 352.7	6.67

1.2 Supplementary Figures

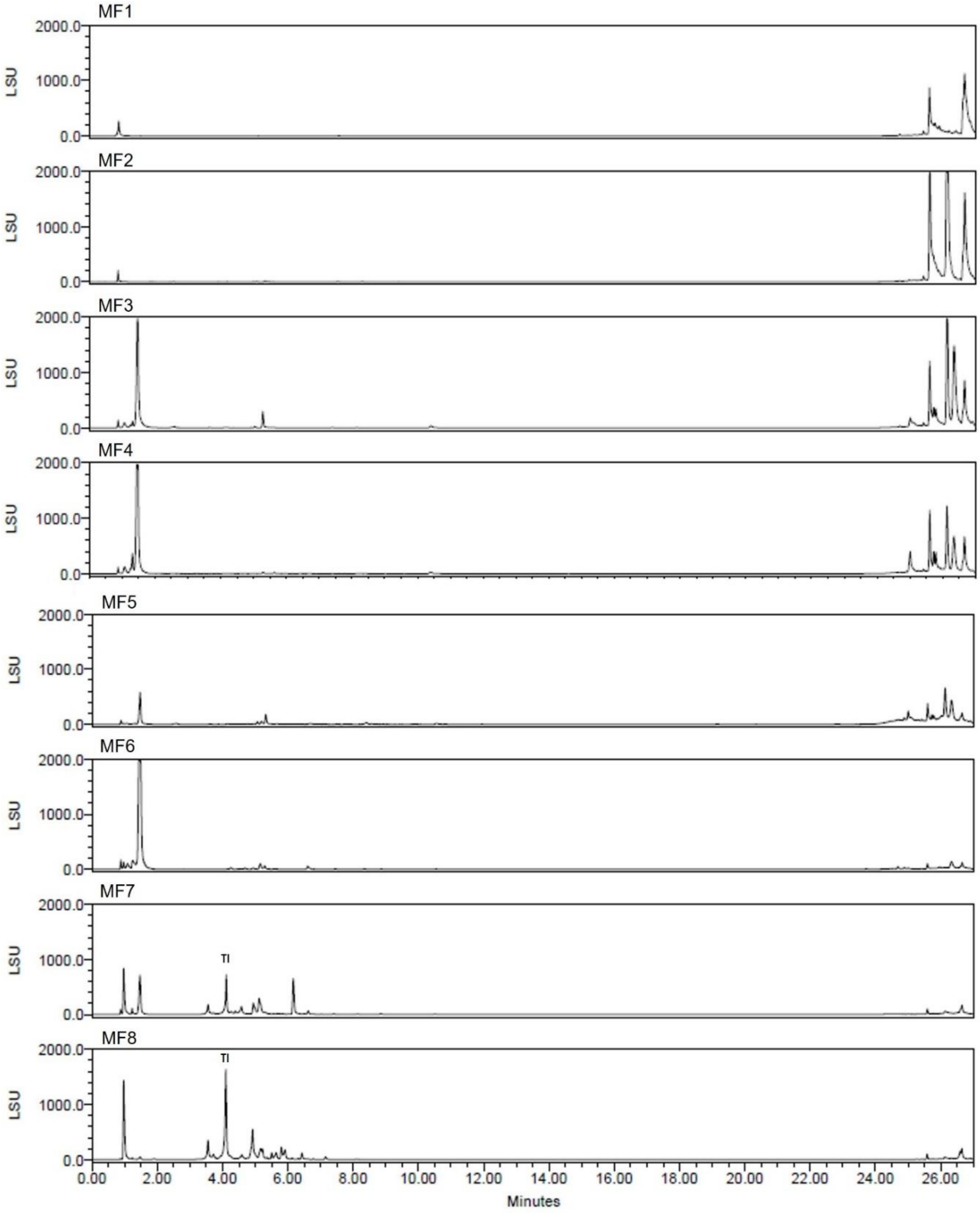

Supplementary Figure 1. UHPLC-ELSD chromatograms of FE-derived microfractions (MFx). Major constituents of FE (summarized in **Supplementary Table 1**) are annotated across the respective MFs. Abbreviations are provided in **Supplementary Table 1** and **Figure 1C**.

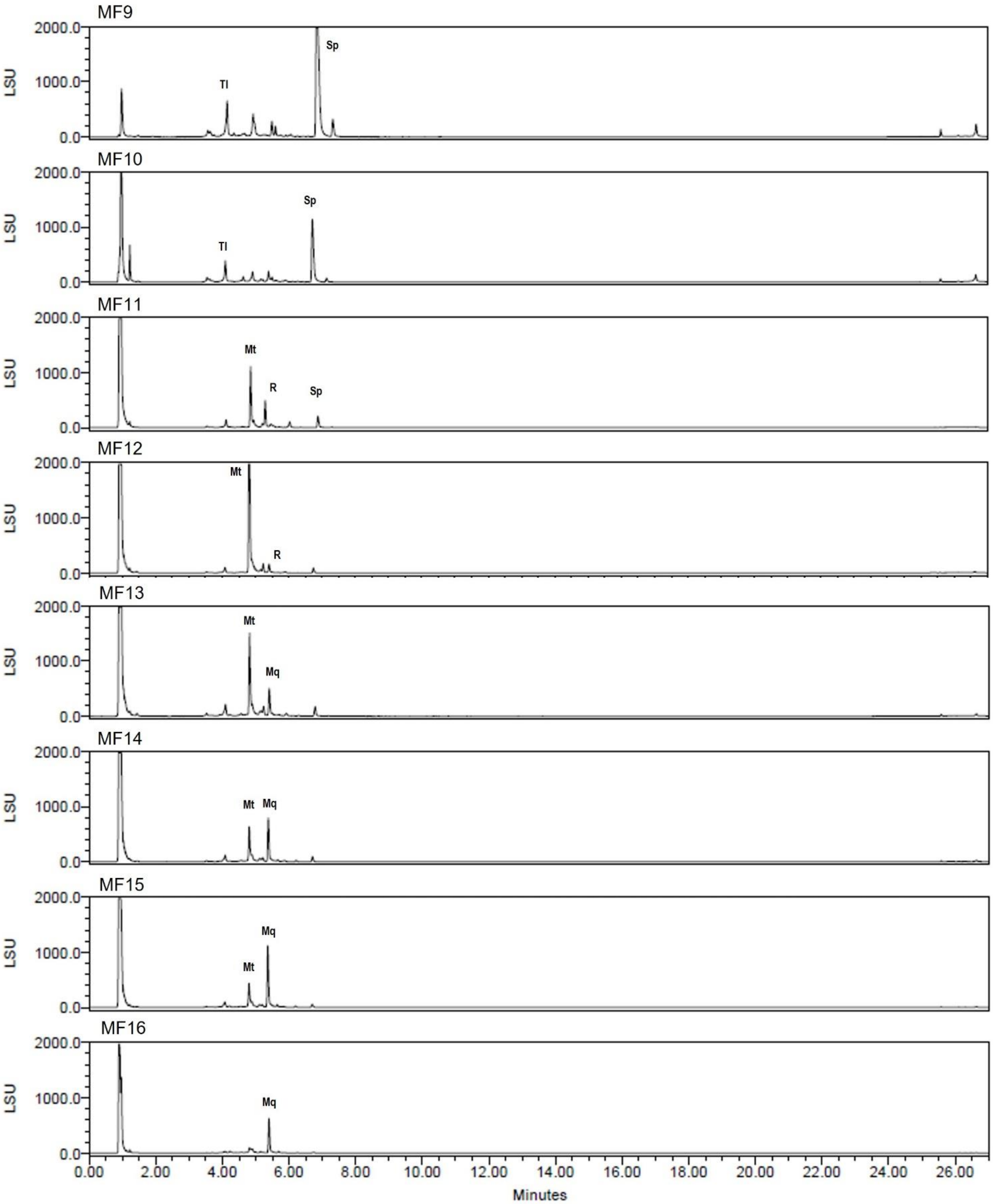

Supplementary Figure 1. Continuing.

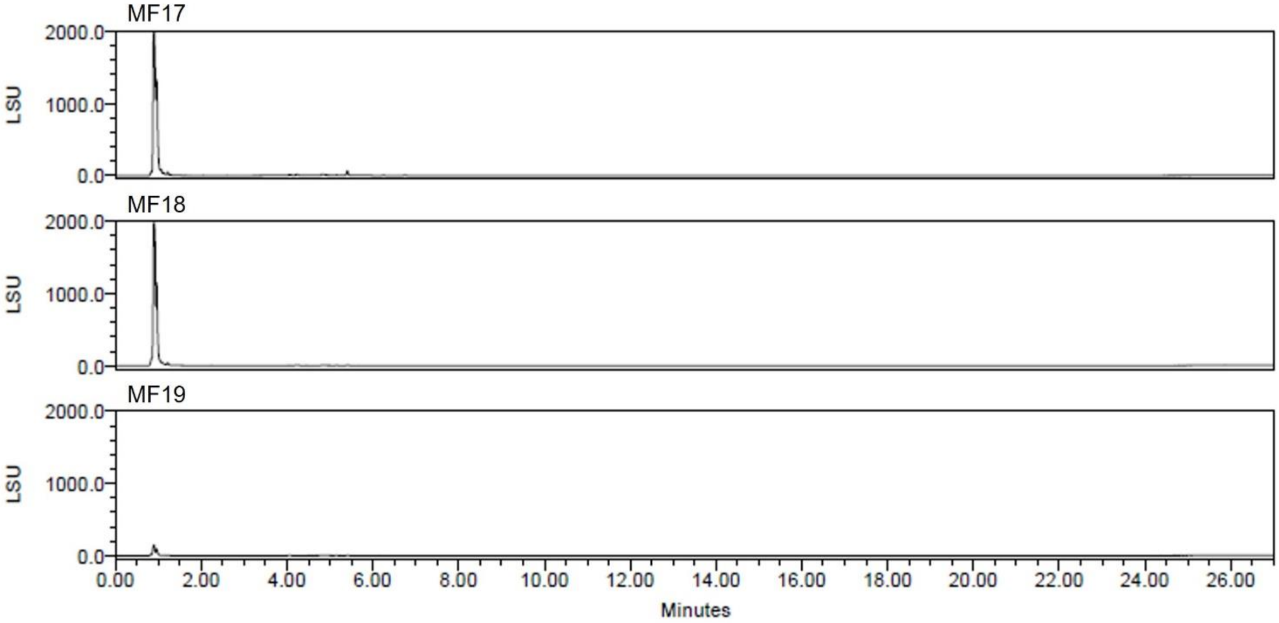

Supplementary Figure 1. Continuing.

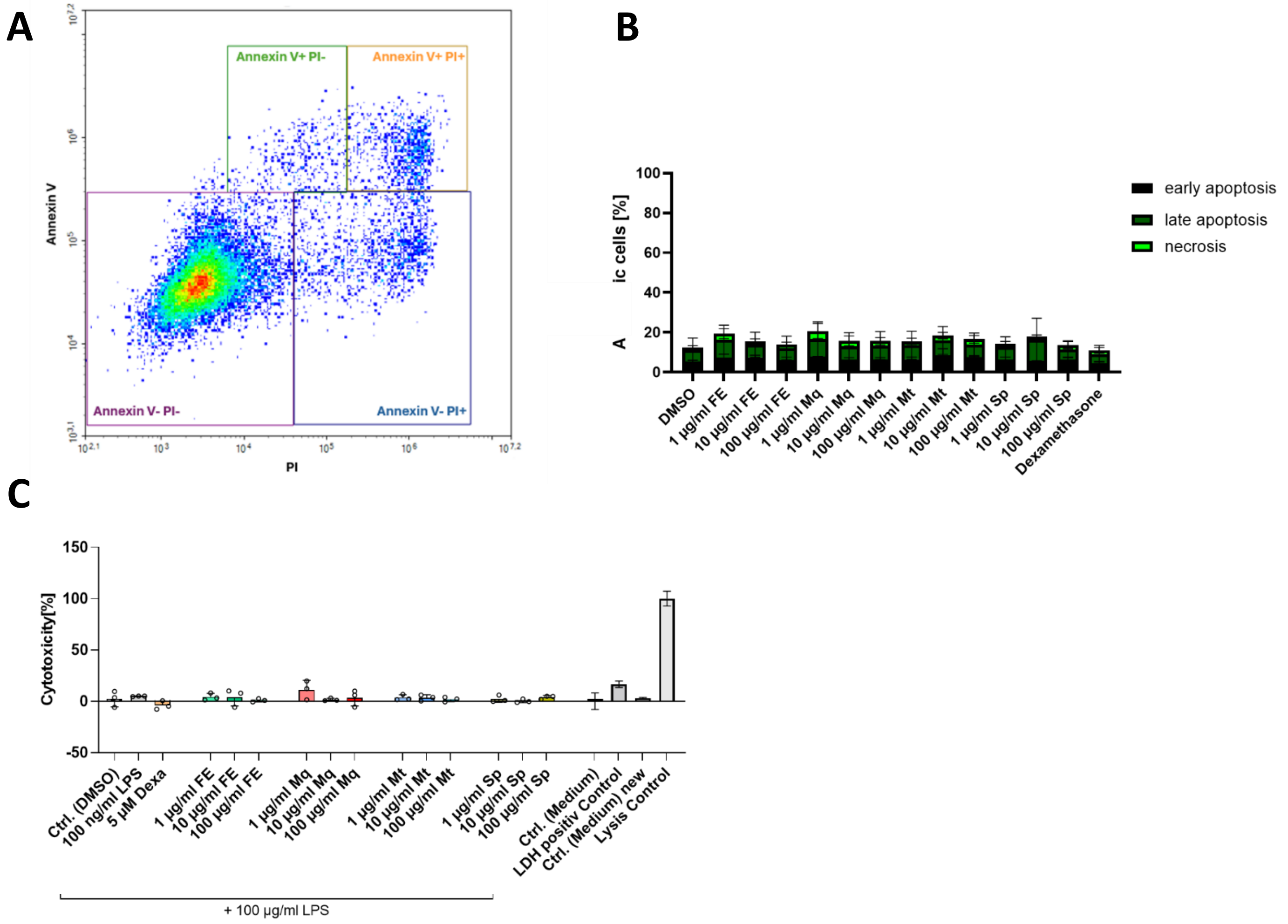

Supplementary Figure 2. Analysis of cytotoxic effects of FE, Mq, Mt, Sp and dexamethasone on HMC3. (**A**) Flow cytometry analysis illustrating the distribution of cells across apoptotic and necrotic stages. Quadrants indicate live cells (Annexin V^{-}/PI^{-}), early apoptosis (Annexin V^{+}/PI^{-}), late apoptosis (Annexin V^{+}/PI^{+}), and necrosis (Annexin V^{-}/PI^{+}). (**B**) Quantification of apoptotic and necrotic cell populations under the treatment with FE, Mq, Mt and Sp (1/ 10/ 100 µg/ml). DMSO (0.1%) treatment represented the effect as soluble control and dexamethasone (5 µM) as positive control in the LPS stimulation assay. Data are presented as percentages of cells in early apoptosis, late apoptosis, and necrosis. Data are means ± SD and individual values from two independent experiments (n=2). (**C**) LDH-assay of supernatants from the HMC3-LPS-stimulation assay after treatment with the controls and FE, Mq, Mt and Sp (1/ 10/ 100 µg/ml). No relevant increases in LDH activity indicating cytotoxicity were found in any of the treatment groups. Data are means ± SD and individual values from one experiment (n=3).

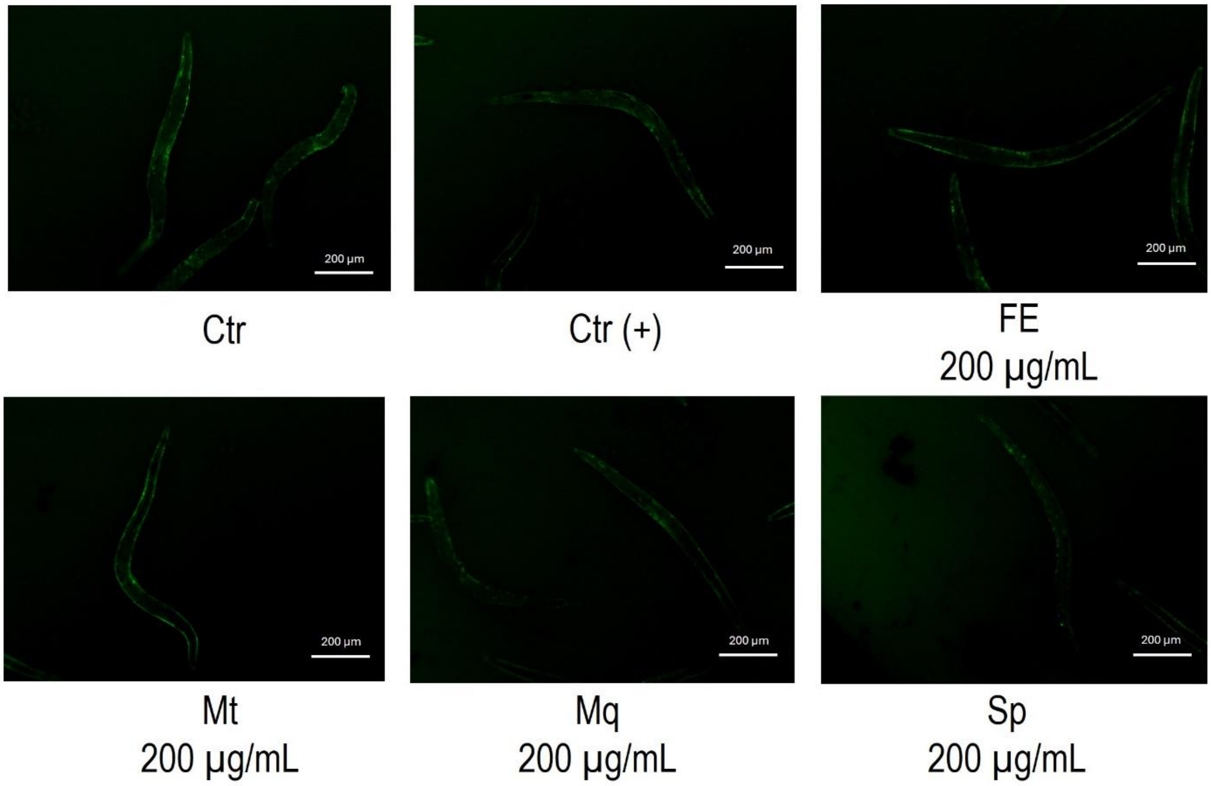

Supplementary Figure 3. Representative fluorescence image of adult transgenic NL5901 *C. elegans* (α-syn::YFP expressed in muscle cells) after being incubated for 5 days with the vehicle control 0.7% DMSO (Ctr), the positive control 2.5 mM levodopa (Ctr(+)) and the samples FE and derived natural compounds monotropitoside (Mt), miquelianin (Mq) and spiraeoside (Sp) at 200 µg/mL. Images were Zeiss Z1 Axio Observer inverted fluorescence microscope equipped with an Axio Cam MRm camera and a GFP filter (excitation 470/40 nm; emission 525/50 nm) under the same exposure setting. Fluorescence is displayed in the green channel.
